# 3-(3-Chloro­phenyl­sulfin­yl)-2,5,7-trimethyl-1-benzofuran

**DOI:** 10.1107/S1600536812008537

**Published:** 2012-03-03

**Authors:** Hong Dae Choi, Pil Ja Seo, Uk Lee

**Affiliations:** a Department of Chemistry, Dongeui University, San 24 Kaya-dong Busanjin-gu, Busan 614-714, Republic of Korea; b Department of Chemistry, Pukyong National University, 599-1 Daeyeon 3-dong, Nam-gu, Busan 608-737, Republic of Korea

## Abstract

In the title compound, C_17_H_15_ClO_2_S, the 3-chloro­phenyl ring makes a dihedral angle of 84.48 (4)° with the mean plane [r.m.s. deviation = 0.004 (1) Å] of the benzofuran fragment. In the crystal, mol­ecules are linked by weak C—H⋯O, C—H⋯π and C—S⋯π [3.414 (2) Å] inter­actions. The crystal structure also exhibits weak π–π inter­actions between the furan rings of neighbouring mol­ecules [centroid–centroid distance = 3.826 (2), inter­planar distance = 3.447 (2) and slippage = 1.660 (2) Å].

## Related literature

For background information and the crystal structures of related compounds, see: Choi *et al.* (2010 ▶, 2011 ▶, 2012 ▶).
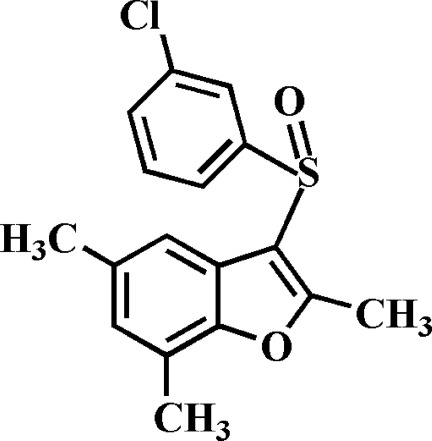


## Experimental

### 

#### Crystal data

C_17_H_15_ClO_2_S
*M* *_r_* = 318.80
Triclinic, 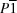

*a* = 6.1701 (1) Å
*b* = 11.6670 (2) Å
*c* = 12.1123 (3) Å
α = 112.965 (1)°
β = 99.114 (1)°
γ = 103.698 (1)°
*V* = 748.70 (3) Å^3^
*Z* = 2
Mo *K*α radiation
μ = 0.40 mm^−1^
*T* = 173 K
0.20 × 0.19 × 0.18 mm

#### Data collection

Bruker SMART APEXII CCD diffractometer
Absorption correction: multi-scan (*SADABS*; Bruker, 2009 ▶) *T*_min_ = 0.926, *T*_max_ = 0.934
13458 measured reflections
3463 independent reflections
3136 reflections with *I* > 2σ(*I*)
*R*_int_ = 0.023

#### Refinement

*R*[*F* ^2^ > 2σ(*F* ^2^)] = 0.034
*wR*(*F* ^2^) = 0.096
*S* = 1.07
3463 reflections
193 parameters
H-atom parameters constrained
Δρ_max_ = 0.29 e Å^−3^
Δρ_min_ = −0.30 e Å^−3^

### 

Data collection: *APEX2* (Bruker, 2009 ▶); cell refinement: *SAINT* (Bruker, 2009 ▶); data reduction: *SAINT*; program(s) used to solve structure: *SHELXS97* (Sheldrick, 2008 ▶); program(s) used to refine structure: *SHELXL97* (Sheldrick, 2008 ▶); molecular graphics: *ORTEP-3* (Farrugia, 1997 ▶) and *DIAMOND* (Brandenburg, 1998 ▶); software used to prepare material for publication: *SHELXL97*.

## Supplementary Material

Crystal structure: contains datablock(s) global, I. DOI: 10.1107/S1600536812008537/lr2053sup1.cif

Structure factors: contains datablock(s) I. DOI: 10.1107/S1600536812008537/lr2053Isup2.hkl

Supplementary material file. DOI: 10.1107/S1600536812008537/lr2053Isup3.cml

Additional supplementary materials:  crystallographic information; 3D view; checkCIF report

## Figures and Tables

**Table 1:** Hydrogen-bond geometry (Å, °) *Cg*1 is the centroid of the C2–C7 benzene ring.

*D*—H⋯*A*	*D*—H	H⋯*A*	*D*⋯*A*	*D*—H⋯*A*
C13—H13⋯O2^i^	0.95	2.55	3.2960 (16)	136
C11—H11*B*⋯*Cg*1^ii^	0.98	2.88	3.703	143

Symmetry codes: (i) 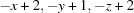; (ii) 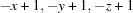.

**Fig. 1.**
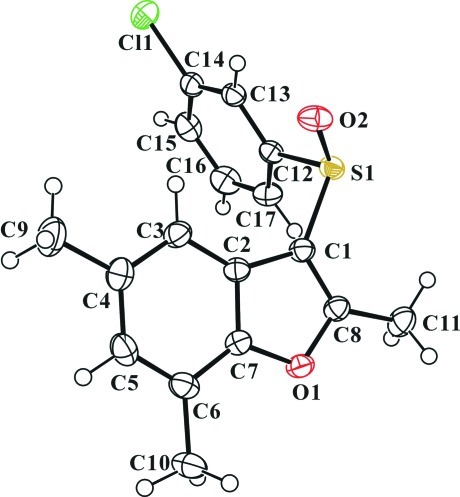
The molecular structure of the title compound. Displacement ellipsoids are drawn at the 50% probability level.

**Fig. 2.**
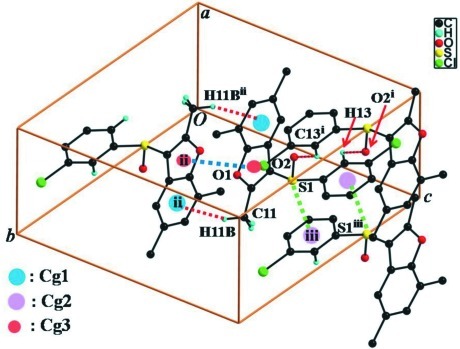
A view of the C—H···O, C—H···π, C—S···π and π–π interactions (dotted lines)in the crystal structure of the title compound. H atoms non-participating in hydrogen-bonding were omitted for clarity. [ Symmetry codes: (i) - x + 2, - y + 1, - z + 2 (ii) - x + 1, - y + 1, - z + 1 (iii) - x + 1, - y + 1 , - z + 2

## supplementary crystallographic information

### Comment

As a part of our ongoing study of 2,5,7-trimethyl-1-benzofuran derivatives containing 3-(4-chlorophenylsulfinyl) (Choi *et al.*,2010), 3-(3-fluorophenylsulfinyl)(Choi *et al.*, 2011) and 3-(4-bromophenylsulfinyl)(Choi *et al.*, 2012) substituents, we report herein the crystal structure of the title compound.

In the title molecule (Fig. 1), the benzofuran unit is essentially planar, with a mean deviation of 0.004 (1) Å from the least-squares plane defined by the nine constituent atoms. The dihedral angle between the 3-chlorobenzene ring and the mean plane of the benzofuran fragment is 84.48 (4)°. The crystal packing (Fig. 2) is stabilized by weak intermolecular C—H···O and C—H···π interactions (Table 1, Cg1 is the centroid of the C2–C7 benzene ring), and by intermolecular S···π interactions between the sulfur atom and the 3-chlorobenzene ring of an adjacent molecule, with S1···Cg2^iii^ being 3.414 (2) Å (Cg2 is the centroid of the C12–C17 3-chlorobenzene ring). Additionally, the crystal packing (Fig. 2) exhibits weak slipped π–π interactions between the furan rings of neighbouring molecules, with a with a Cg3···Cg3^ii^ distance of 3.826 (2) Å and an interplanar distance of 3.447 (2) Å resulting in a slippage of 1.660 (2) Å (Cg3 is the centroid of the C1/C2/C7/O1/C8 furan ring).

### Experimental

77% 3-Chloroperoxybenzoic acid (247 mg, 1.1 mmol) was added in small portions to a stirred solution of 3-(3-chlorophenylsulfanyl)-2,5,7-trimethyl-1-benzofuran (303 mg, 1.0 mmol) in dichloromethane (30 mL) at 273 K. After being stirred at room temperature for 4h, the mixture was washed with saturated sodium bicarbonate solution and the organic layer was separated, dried over magnesium sulfate, filtered and concentrated at reduced pressure. The residue was purified by column chromatography (hexane–ethyl acetate, 2:1 v/v) to afford the title compound as a colorless solid [yield 78%, m.p. 428–429 K; *R*_f_ = 0.49 (hexane–ethyl acetate, 2:1 v/v)]. Single crystals suitable for X-ray diffraction were prepared by slow evaporation of a solution of the title compound in ethyl acetate at room temperature.

### Refinement

All H atoms were positioned geometrically and refined using a riding model, with C—H = 0.95 Å for aryl and 0.98 Å for methyl H atoms. *U*_iso_(H) =1.2*U*_eq_(C) for aryl and 1.5*U*_eq_(C)for methyl H atoms.

### Crystal data

**Table:**

C_17_H_15_ClO_2_S	*Z* = 2
*M**_r_* = 318.80	*F*(000) = 332
Triclinic, *P*1	*D*_x_ = 1.414 Mg m^−^^3^
Hall symbol: -P 1	Mo *K*α radiation, λ = 0.71073 Å
*a* = 6.1701 (1) Å	Cell parameters from 7460 reflections
*b* = 11.6670 (2) Å	θ = 3.3–27.6°
*c* = 12.1123 (3) Å	µ = 0.40 mm^−^^1^
α = 112.965 (1)°	*T* = 173 K
β = 99.114 (1)°	Block, colourless
γ = 103.698 (1)°	0.20 × 0.19 × 0.18 mm
*V* = 748.70 (3) Å^3^	

### Data collection

**Table:**

Bruker SMART APEXII CCD diffractometer	3463 independent reflections
Radiation source: rotating anode	3136 reflections with *I* > 2σ(*I*)
Graphite multilayer monochromator	*R*_int_ = 0.023
Detector resolution: 10.0 pixels mm^-1^	θ_max_ = 27.6°, θ_min_ = 1.9°
φ and ω scans	*h* = −7→8
Absorption correction: multi-scan (*SADABS*; Bruker, 2009)	*k* = −15→15
*T*_min_ = 0.926, *T*_max_ = 0.934	*l* = −14→15
13458 measured reflections	

### Refinement

**Table:**

Refinement on *F*^2^	Primary atom site location: structure-invariant direct methods
Least-squares matrix: full	Secondary atom site location: difference Fourier map
*R*[*F*^2^ > 2σ(*F*^2^)] = 0.034	Hydrogen site location: difference Fourier map
*wR*(*F*^2^) = 0.096	H-atom parameters constrained
*S* = 1.07	*w* = 1/[σ^2^(*F*_o_^2^) + (0.0531*P*)^2^ + 0.2623*P*] where *P* = (*F*_o_^2^ + 2*F*_c_^2^)/3
3463 reflections	(Δ/σ)_max_ = 0.001
193 parameters	Δρ_max_ = 0.29 e Å^−^^3^
0 restraints	Δρ_min_ = −0.30 e Å^−^^3^

### Special details

**Table:**

Geometry. All esds (except the esd in the dihedral angle between two l.s. planes) are estimated using the full covariance matrix. The cell esds are taken into account individually in the estimation of esds in distances, angles and torsion angles; correlations between esds in cell parameters are only used when they are defined by crystal symmetry. An approximate (isotropic) treatment of cell esds is used for estimating esds involving l.s. planes.
Refinement. Refinement of F^2^ against ALL reflections. The weighted R-factor wR and goodness of fit S are based on F^2^, conventional R-factors R are based on F, with F set to zero for negative F^2^. The threshold expression of F^2^ > 2sigma(F^2^) is used only for calculating R-factors(gt) etc. and is not relevant to the choice of reflections for refinement. R-factors based on F^2^ are statistically about twice as large as those based on F, and R- factors based on ALL data will be even larger.

### Fractional atomic coordinates and isotropic or equivalent isotropic displacement parameters (Å^2^)

**Table:**

	*x*	*y*	*z*	*U*_iso_*/*U*_eq_	
Cl1	0.71046 (7)	0.15422 (4)	1.01673 (4)	0.03980 (13)	
S1	0.62148 (6)	0.50385 (3)	0.83504 (3)	0.02455 (11)	
O1	0.24560 (17)	0.38716 (10)	0.49273 (9)	0.0276 (2)	
O2	0.87230 (17)	0.51728 (11)	0.85352 (10)	0.0334 (2)	
C1	0.4848 (2)	0.42875 (13)	0.67382 (12)	0.0240 (3)	
C2	0.5205 (2)	0.32070 (13)	0.57651 (12)	0.0236 (3)	
C3	0.6609 (2)	0.24231 (14)	0.57050 (14)	0.0286 (3)	
H3	0.7670	0.2559	0.6440	0.034*	
C4	0.6425 (3)	0.14386 (15)	0.45483 (15)	0.0328 (3)	
C5	0.4846 (3)	0.12593 (15)	0.34719 (14)	0.0338 (3)	
H5	0.4745	0.0580	0.2689	0.041*	
C6	0.3429 (3)	0.20295 (15)	0.34993 (13)	0.0303 (3)	
C7	0.3683 (2)	0.29925 (14)	0.46700 (13)	0.0248 (3)	
C8	0.3205 (2)	0.46457 (14)	0.61921 (13)	0.0253 (3)	
C9	0.7918 (3)	0.05666 (19)	0.44441 (19)	0.0455 (4)	
H9A	0.9141	0.0814	0.4073	0.068*	
H9B	0.6954	−0.0356	0.3914	0.068*	
H9C	0.8628	0.0675	0.5277	0.068*	
C10	0.1747 (3)	0.18530 (18)	0.23546 (15)	0.0428 (4)	
H10A	0.0170	0.1662	0.2447	0.064*	
H10B	0.1809	0.1119	0.1618	0.064*	
H10C	0.2166	0.2663	0.2252	0.064*	
C11	0.2052 (3)	0.56436 (16)	0.66873 (15)	0.0338 (3)	
H11A	0.0457	0.5201	0.6645	0.051*	
H11B	0.2023	0.6129	0.6186	0.051*	
H11C	0.2914	0.6259	0.7557	0.051*	
C12	0.4908 (2)	0.36608 (13)	0.86309 (12)	0.0234 (3)	
C13	0.6366 (2)	0.31970 (13)	0.92070 (12)	0.0245 (3)	
H13	0.8008	0.3577	0.9432	0.029*	
C14	0.5339 (3)	0.21551 (14)	0.94437 (13)	0.0275 (3)	
C15	0.2958 (3)	0.16024 (15)	0.91376 (14)	0.0335 (3)	
H15	0.2296	0.0886	0.9305	0.040*	
C16	0.1544 (3)	0.21071 (16)	0.85821 (15)	0.0361 (3)	
H16	−0.0097	0.1734	0.8368	0.043*	
C17	0.2507 (3)	0.31488 (15)	0.83378 (14)	0.0311 (3)	
H17	0.1541	0.3508	0.7975	0.037*	

### Atomic displacement parameters (Å^2^)

**Table:**

	*U*^11^	*U*^22^	*U*^33^	*U*^12^	*U*^13^	*U*^23^
Cl1	0.0494 (2)	0.0350 (2)	0.0403 (2)	0.01686 (18)	0.00730 (17)	0.02221 (18)
S1	0.02701 (19)	0.02439 (18)	0.01981 (17)	0.00680 (13)	0.00449 (12)	0.00919 (13)
O1	0.0276 (5)	0.0334 (5)	0.0249 (5)	0.0140 (4)	0.0052 (4)	0.0146 (4)
O2	0.0237 (5)	0.0406 (6)	0.0315 (5)	0.0026 (4)	0.0024 (4)	0.0184 (5)
C1	0.0247 (6)	0.0269 (6)	0.0217 (6)	0.0098 (5)	0.0062 (5)	0.0114 (5)
C2	0.0233 (6)	0.0262 (7)	0.0222 (6)	0.0081 (5)	0.0066 (5)	0.0116 (5)
C3	0.0268 (7)	0.0320 (7)	0.0301 (7)	0.0133 (6)	0.0072 (5)	0.0152 (6)
C4	0.0341 (8)	0.0313 (7)	0.0379 (8)	0.0150 (6)	0.0150 (6)	0.0158 (6)
C5	0.0425 (9)	0.0281 (7)	0.0271 (7)	0.0111 (6)	0.0129 (6)	0.0076 (6)
C6	0.0328 (7)	0.0315 (7)	0.0232 (7)	0.0068 (6)	0.0056 (6)	0.0118 (6)
C7	0.0249 (6)	0.0271 (6)	0.0246 (6)	0.0097 (5)	0.0067 (5)	0.0131 (5)
C8	0.0267 (6)	0.0284 (7)	0.0247 (6)	0.0111 (5)	0.0089 (5)	0.0139 (5)
C9	0.0517 (10)	0.0430 (9)	0.0514 (10)	0.0295 (8)	0.0222 (8)	0.0195 (8)
C10	0.0516 (10)	0.0425 (9)	0.0239 (7)	0.0109 (8)	−0.0015 (7)	0.0117 (7)
C11	0.0350 (8)	0.0372 (8)	0.0380 (8)	0.0207 (7)	0.0144 (6)	0.0186 (7)
C12	0.0257 (6)	0.0246 (6)	0.0180 (6)	0.0077 (5)	0.0064 (5)	0.0080 (5)
C13	0.0244 (6)	0.0257 (6)	0.0210 (6)	0.0082 (5)	0.0056 (5)	0.0084 (5)
C14	0.0355 (7)	0.0254 (6)	0.0211 (6)	0.0117 (6)	0.0073 (5)	0.0092 (5)
C15	0.0382 (8)	0.0283 (7)	0.0310 (7)	0.0046 (6)	0.0127 (6)	0.0129 (6)
C16	0.0251 (7)	0.0394 (8)	0.0380 (8)	0.0037 (6)	0.0094 (6)	0.0154 (7)
C17	0.0249 (7)	0.0383 (8)	0.0292 (7)	0.0096 (6)	0.0056 (5)	0.0154 (6)

### Geometric parameters (Å, º)

**Table:**

Cl1—C14	1.7385 (15)	C9—H9A	0.9800
S1—O2	1.4892 (11)	C9—H9B	0.9800
S1—C1	1.7564 (14)	C9—H9C	0.9800
S1—C12	1.8023 (14)	C10—H10A	0.9800
O1—C8	1.3692 (17)	C10—H10B	0.9800
O1—C7	1.3839 (17)	C10—H10C	0.9800
C1—C8	1.3577 (19)	C11—H11A	0.9800
C1—C2	1.4427 (18)	C11—H11B	0.9800
C2—C3	1.3910 (19)	C11—H11C	0.9800
C2—C7	1.3924 (18)	C12—C13	1.3840 (19)
C3—C4	1.388 (2)	C12—C17	1.3880 (19)
C3—H3	0.9500	C13—C14	1.388 (2)
C4—C5	1.407 (2)	C13—H13	0.9500
C4—C9	1.510 (2)	C14—C15	1.379 (2)
C5—C6	1.390 (2)	C15—C16	1.388 (2)
C5—H5	0.9500	C15—H15	0.9500
C6—C7	1.379 (2)	C16—C17	1.383 (2)
C6—C10	1.504 (2)	C16—H16	0.9500
C8—C11	1.485 (2)	C17—H17	0.9500
			
O2—S1—C1	108.33 (6)	H9A—C9—H9C	109.5
O2—S1—C12	105.99 (6)	H9B—C9—H9C	109.5
C1—S1—C12	96.82 (6)	C6—C10—H10A	109.5
C8—O1—C7	106.60 (10)	C6—C10—H10B	109.5
C8—C1—C2	107.66 (12)	H10A—C10—H10B	109.5
C8—C1—S1	124.30 (11)	C6—C10—H10C	109.5
C2—C1—S1	128.05 (10)	H10A—C10—H10C	109.5
C3—C2—C7	119.35 (13)	H10B—C10—H10C	109.5
C3—C2—C1	136.00 (13)	C8—C11—H11A	109.5
C7—C2—C1	104.65 (12)	C8—C11—H11B	109.5
C4—C3—C2	118.57 (13)	H11A—C11—H11B	109.5
C4—C3—H3	120.7	C8—C11—H11C	109.5
C2—C3—H3	120.7	H11A—C11—H11C	109.5
C3—C4—C5	119.72 (14)	H11B—C11—H11C	109.5
C3—C4—C9	120.17 (15)	C13—C12—C17	121.99 (13)
C5—C4—C9	120.11 (15)	C13—C12—S1	118.00 (10)
C6—C5—C4	123.12 (14)	C17—C12—S1	119.90 (11)
C6—C5—H5	118.4	C12—C13—C14	117.46 (13)
C4—C5—H5	118.4	C12—C13—H13	121.3
C7—C6—C5	114.77 (13)	C14—C13—H13	121.3
C7—C6—C10	121.63 (15)	C15—C14—C13	122.02 (14)
C5—C6—C10	123.60 (15)	C15—C14—Cl1	118.97 (12)
C6—C7—O1	125.10 (13)	C13—C14—Cl1	119.01 (11)
C6—C7—C2	124.46 (13)	C14—C15—C16	119.08 (14)
O1—C7—C2	110.43 (12)	C14—C15—H15	120.5
C1—C8—O1	110.66 (12)	C16—C15—H15	120.5
C1—C8—C11	133.42 (13)	C17—C16—C15	120.50 (14)
O1—C8—C11	115.88 (12)	C17—C16—H16	119.8
C4—C9—H9A	109.5	C15—C16—H16	119.8
C4—C9—H9B	109.5	C16—C17—C12	118.90 (14)
H9A—C9—H9B	109.5	C16—C17—H17	120.6
C4—C9—H9C	109.5	C12—C17—H17	120.6
			
O2—S1—C1—C8	−139.54 (12)	C1—C2—C7—C6	−179.32 (13)
C12—S1—C1—C8	111.08 (13)	C3—C2—C7—O1	−179.80 (12)
O2—S1—C1—C2	40.95 (14)	C1—C2—C7—O1	0.13 (15)
C12—S1—C1—C2	−68.43 (13)	C2—C1—C8—O1	0.15 (16)
C8—C1—C2—C3	179.75 (16)	S1—C1—C8—O1	−179.45 (10)
S1—C1—C2—C3	−0.7 (2)	C2—C1—C8—C11	177.69 (15)
C8—C1—C2—C7	−0.17 (15)	S1—C1—C8—C11	−1.9 (2)
S1—C1—C2—C7	179.41 (11)	C7—O1—C8—C1	−0.07 (15)
C7—C2—C3—C4	−0.6 (2)	C7—O1—C8—C11	−178.08 (12)
C1—C2—C3—C4	179.46 (15)	O2—S1—C12—C13	14.09 (12)
C2—C3—C4—C5	0.2 (2)	C1—S1—C12—C13	125.42 (11)
C2—C3—C4—C9	−179.98 (15)	O2—S1—C12—C17	−169.67 (11)
C3—C4—C5—C6	0.1 (2)	C1—S1—C12—C17	−58.34 (12)
C9—C4—C5—C6	−179.69 (15)	C17—C12—C13—C14	2.4 (2)
C4—C5—C6—C7	0.0 (2)	S1—C12—C13—C14	178.57 (10)
C4—C5—C6—C10	179.59 (16)	C12—C13—C14—C15	−0.8 (2)
C5—C6—C7—O1	−179.78 (13)	C12—C13—C14—Cl1	−179.94 (10)
C10—C6—C7—O1	0.6 (2)	C13—C14—C15—C16	−0.4 (2)
C5—C6—C7—C2	−0.4 (2)	Cl1—C14—C15—C16	178.74 (12)
C10—C6—C7—C2	179.97 (14)	C14—C15—C16—C17	0.0 (2)
C8—O1—C7—C6	179.40 (13)	C15—C16—C17—C12	1.5 (2)
C8—O1—C7—C2	−0.04 (15)	C13—C12—C17—C16	−2.7 (2)
C3—C2—C7—C6	0.8 (2)	S1—C12—C17—C16	−178.83 (12)

### Hydrogen-bond geometry (Å, º)

Cg1 is the centroid of the C2–C7 benzene ring.

**Table:**

*D*—H···*A*	*D*—H	H···*A*	*D*···*A*	*D*—H···*A*
C13—H13···O2^i^	0.95	2.55	3.2960 (16)	136
C11—H11*B*···*Cg*1^ii^	0.98	2.88	3.703	143

Symmetry codes: (i) −*x*+2, −*y*+1, −*z*+2; (ii) −*x*+1, −*y*+1, −*z*+1.
